# Differential Causal Associations of Gut Microbiota, Blood Metabolites, and Immune Cell Phenotypes With Early- and Late-Onset Alzheimer’s Disease: A Bidirectional Mendelian Randomization Analysis

**DOI:** 10.7759/cureus.109543

**Published:** 2026-05-24

**Authors:** Yuejun Wang, LIfang Wang, Dehua Zhan

**Affiliations:** 1 Department of Geriatrics, Zhejiang Aged Care Hospital, Hangzhou Normal University, Hangzhou, CHN

**Keywords:** alzheimer's disease subtypes, bidirectional mendelian randomization, blood metabolites, early-onset alzheimer's disease, gut microbiota, gut microbiota-immune-metabolic axis, immune cell phenotypes, late-onset alzheimer's disease

## Abstract

Background: Alzheimer’s disease (AD) is a heterogeneous syndrome with distinct genetic and clinical profiles between early-onset AD (EOAD) and late-onset AD (LOAD) subtypes. However, specific causal etiologies linking the gut microbiota-immune-metabolic axis to these subtypes remain poorly understood.

Methods: We employed a comprehensive bidirectional two-sample Mendelian randomization (MR) framework to systematically investigate the causal associations of gut microbiota, immune cell phenotypes, and blood metabolites with EOAD and LOAD. Large-scale genome-wide association study (GWAS) summary statistics were utilized from the MiBioGen consortium, Sardinian cohort, and Canadian Longitudinal Study on Aging, alongside AD outcome data from the FinnGen consortium. Causal estimates were generated using the inverse variance-weighted method, with rigorous sensitivity analyses including false discovery rate (FDR) correction and Steiger directionality tests to ensure robustness.

Results: Our analysis revealed divergent multi-omics signatures for AD subtypes. While the genus *Veillonella* and myeloid dendritic cells emerged as shared protective factors, the risk profiles were distinct. EOAD susceptibility was primarily driven by adaptive immune dysregulation and lipid metabolism disturbances. In contrast, LOAD risk was predominantly associated with innate immune dysfunction and perturbations in amino acid and gut-derived metabolite turnover, such as hippurate.

Conclusions: This study provides genetic evidence that EOAD and LOAD are driven by fundamentally different peripheral mechanisms across the gut-immune-metabolic axis. These findings challenge the monolithic view of AD pathogenesis and underscore the critical necessity of stratifying patients by onset age to develop precision therapeutic interventions.

## Introduction

Alzheimer's disease (AD) represents a burgeoning global health crisis, currently affecting over 55 million individuals worldwide, with projections estimating a rise to 139 million by 2050 [1]. Beyond the staggering epidemiological figures, the economic burden is projected to exceed $2.8 trillion by 2030, necessitating urgent identification of modifiable risk factors and therapeutic targets. Clinically and etiologically, AD is not a monolithic entity but rather a heterogeneous syndrome. A critical yet often overlooked distinction lies between early-onset AD (EOAD) (onset <65 years) and late-onset AD (LOAD) (onset ≥65 years). While they share core neuropathological hallmarks-amyloid-beta (Aβ) plaques and tau neurofibrillary tangles, their genetic architectures and progression trajectories diverge significantly [2].

EOAD, accounting for approximately 5-10% of cases, is frequently driven by high-penetrance mutations in *APP*, *PSEN1*, or *PSEN2; *however*, *a substantial portion of EOAD variance remains unexplained by these Mendelian mutations, suggesting a complex polygenic etiology involving distinct metabolic and immune pathways [3]. Conversely, LOAD is predominantly sporadic, heavily influenced by the APOE ε4 allele and aging-related systemic dysregulation [4]. Despite these marked differences, the vast majority of existing etiological research, particularly large-scale omics studies, has conflated these subtypes or focused exclusively on LOAD. This reductionist approach potentially obscures subtype-specific pathophysiological mechanisms, particularly those involving the dynamic interplay between peripheral systems and the central nervous system (CNS).

Emerging evidence over the past decade has positioned the gut microbiota as a pivotal regulator of brain function via the "microbiota-gut-brain axis" [5]. Observational cross-sectional and cohort studies published between 2019 and 2025 have consistently documented dysbiosis in AD patients, characterized by a decrease in anti-inflammatory taxa (e.g., *Faecalibacterium prausnitzii*, *Eubacterium rectale*) and an enrichment of pro-inflammatory genera (e.g., *Escherichia*/*Shigella*, *Bacteroides*) [6]. Mechanistically, it is hypothesized that gut dysbiosis exacerbates AD pathology through multiple pathways: increasing intestinal permeability ("leaky gut"), facilitating the translocation of bacterial endotoxins (lipopolysaccharides) into the systemic circulation, and modulating microglial activation state within the brain [7]. However, the interpretation of these observational findings is severely limited by the potential for reverse causation and unmeasured confounding. For instance, dietary changes or medication use in symptomatic AD patients may alter the microbiome, creating a "chicken-or-egg" dilemma [8]. To address this, recent Mendelian randomization (MR) studies have utilized genetic variants as instrumental variables to infer causality. While recent work has identified causal links between specific gut traits and AD risk [9], few have systematically investigated whether these microbial causal effects differ between EOAD and LOAD. Furthermore, the directionality of these associations remains under-explored; it is plausible that AD-related neurodegeneration itself alters gut physiology, necessitating a bidirectional analytical framework.

The crosstalk between the gut and the brain is largely mediated by circulating metabolites, which serve as the functional readout of microbial activity and host metabolism [10]. Gut microbiota-derived metabolites, such as short-chain fatty acids (SCFAs), bile acids, and tryptophan catabolites, can cross the blood-brain barrier (BBB) and directly influence neuronal signaling and synaptic plasticity [11]. Recent metabolomic profiling has identified perturbations in lipid metabolism (e.g., sphingolipids, phosphatidylcholines) and amino acid pathways (e.g., glutamine, branched-chain amino acids) in AD patients [12]. Crucially, a 2024 MR study utilizing extensive GWAS data highlighted a protective causal role of circulating glutamine in LOAD, suggesting that specific metabolic signatures may confer resilience against neurodegeneration [13]. Nevertheless, the metabolic landscape of EOAD remains poorly characterized in comparison to LOAD. Investigating the causal metabolic networks distinct to each subtype is essential, as EOAD patients often present with different systemic metabolic profiles, potentially linked to faster disease progression [14]. Understanding whether specific metabolites act as mediators between gut dysbiosis and AD subtypes could unveil precision nutrition strategies or pharmacological interventions tailored to disease onset age.

Concurrent with metabolic dysregulation, the immune system plays a dual role in AD pathogenesis. The "neuroinflammation hypothesis" posits that sustained activation of microglia and astrocytes drives synaptic loss and neuronal death [15]. However, this process is not confined to the CNS; peripheral immune dynamics, including the ratio of lymphocytes to monocytes and the expression of specific surface antigens on T-cells, significantly influence central inflammatory states [16]. Recent GWAS and MR analyses have begun to map the causal effects of immune cell traits on AD. For instance, specific immunophenotypes, such as the absolute count of CD4+ T cells or the expression of chemokine receptors on monocytes, have been causally linked to AD risk [17]. Yet, a significant knowledge gap persists regarding the "immune-microbiome-metabolite" triad. The immune system likely acts as both a responder to gut dysbiosis and an effector of neurodegeneration. To date, no study has simultaneously modeled the causal architecture of immune phenotypes in the context of microbiome and metabolome variations, specifically stratifying by AD onset age.

To address the aforementioned gaps, we conducted a bidirectional MR study to systematically explore and compare the differential causal associations of gut microbiota, blood metabolites, and immune cell phenotypes with EOAD and LOAD. We hypothesized that: (i) gut microbial profiles would differ causally between EOAD and LOAD, with distinct taxa contributing to subtype-specific risk, (ii) immune phenotypes would show divergent causal links, with adaptive immunity more relevant to EOAD and innate immunity to LOAD, and (iii) metabolic profiles would differ, with lipid metabolism dominating EOAD while amino acid and gut-derived metabolites dominate LOAD. This study aims to provide novel insights into the differential pathogenesis of EOAD and LOAD and identify potential peripheral therapeutic targets tailored to AD subtypes.

## Materials and methods

Study design and framework

**Figure 1:**
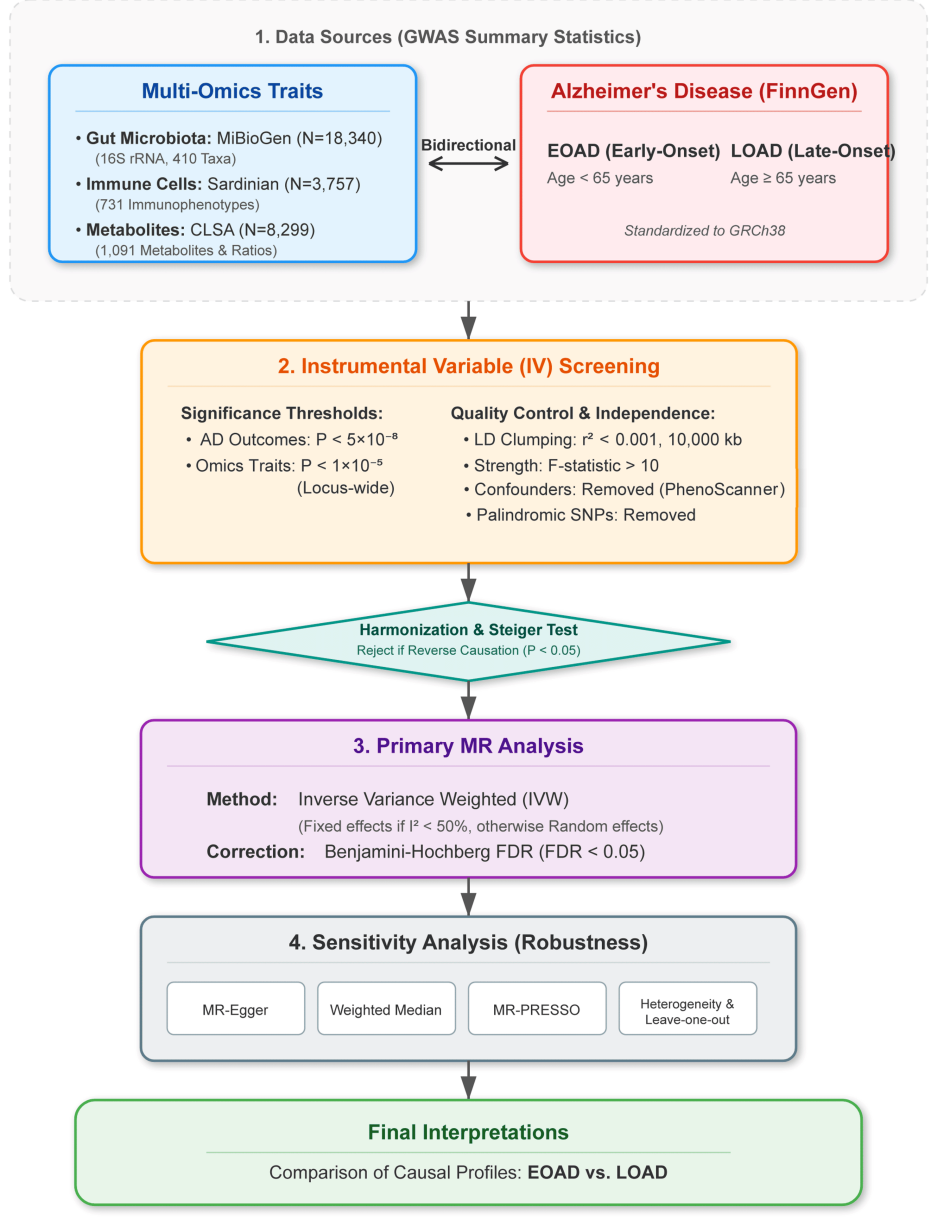
Bidirectional Mendelian randomization framework integrating GWAS summary statistics for gut microbiota, metabolites, and immune phenotypes with EOAD and LOAD data CLSA: Canadian Longitudinal Study on Aging; GWAS: genome-wide association study; EOAD: early-onset Alzheimer’s disease; LOAD: late-onset Alzheimer’s disease; AD: Alzheimer’s disease; LD: linkage disequilibrium; SNP: single nucleotide polymorphism; MR: Mendelian randomization; FDR: false discovery rate; MR-PRESSO: Mendelian Randomization Pleiotropy RESidual Sum and Outlier

A bidirectional two-sample MR framework was employed to systematically interrogate the causal associations and potential heterogeneities of gut microbiota, serum immune cell phenotypes, and circulating metabolites with EOAD and LOAD. To ensure the validity of causal inference, this study was strictly designed to satisfy the three core assumptions of MR: (i) the genetic variants utilized as IVs must be robustly associated with the exposure of interest, (2) the IVs must be independent of potential confounding factors, and (iii) the IVs must influence the outcome solely through the exposure pathway, thereby excluding horizontal pleiotropy. The analysis followed a closed-loop workflow encompassing data harmonization, rigorous IV screening, bidirectional causal estimation, and comprehensive sensitivity validation (Figure 1).

Data sources for exposures and outcomes

Summary statistics for the exposures were obtained from large-scale genome-wide association studies (GWAS), predominantly involving individuals of European ancestry to minimize population stratification bias. Gut microbiota genetic data were obtained from the MiBioGen consortium meta-analysis (N=18,340), which harmonized 16S rRNA V4 region sequencing data across 24 cohorts, covering 410 genus-level taxonomic units [18]. Raw reads were processed using the QIIME2 pipeline (https://qiime2.org/), and taxonomic classification was performed against the SILVA 132 database at 97% sequence identity [19]. Immune cell phenotype data were derived from the Sardinian cohort (N=3,757) of the study by Orrù et al., including 731 immunophenotypes (absolute counts, median fluorescence intensities, morphological parameters) [20]. In their study, measurements were performed via standardized 10-color flow cytometry on fresh peripheral blood, with hierarchical gating, normalization, and batch correction to minimize technical variation. Metabolic profiles were sourced from the Canadian Longitudinal Study on Aging (CLSA) cohort (N=8,299), providing 1,091 unique plasma metabolites and 309 metabolite ratios [21]. Profiling used non-targeted liquid chromatography-mass spectrometry (LC-MS) in both positive and negative ionization modes, with identification confirmed against an in-house authentic standard library and quantitative accuracy ensured by internal standards and pooled quality controls.

Regarding the outcomes, genetic summary statistics for AD were acquired from the FinnGen consortium (Release 12) [22]. To dissect the age-dependent etiology of AD, cases were stratified into two distinct subgroups based on age of onset: EOAD (onset age < 65 years, 1820 cases and 216,472 controls) and LOAD (onset age ≥ 65 years, 9690 cases and 216,472 controls), defined according to ICD-10 code G30 [23].

To prevent model overfitting due to sample overlap, we prioritized the use of independent, non-overlapping European populations between exposure and outcome datasets. All genomic coordinates were standardized to the GRCh38 reference genome, and allele directions were harmonized using strand flipping where necessary. MiBioGen, the Sardinian cohort, and CLSA have no sample overlap with the Finland-only FinnGen consortium, mitigating sample overlap bias in our two-sample MR analyses.

Selection and validation of IVs

To construct robust genetic instruments, we applied a hierarchical screening strategy tailored to the specific characteristics of each dataset. For EOAD and LOAD as exposures, we selected single-nucleotide polymorphisms (SNPs) reaching the conventional genome-wide significance threshold (P < 5×10-8). Conversely, for gut microbiota, immune phenotypes, and metabolites, traits that typically yield fewer signals at genome-wide levels due to sample size constraints, we adopted a locus-wide significance threshold (P < 1×10-5) to ensure a sufficient number of IVs for analysis. While relaxing the P-value threshold aims to maintain statistical power, it inherently increases the potential risk of weak instrument bias and multiplicity. To strictly mitigate these risks, we subsequently calculated the F-statistic for each IV. To guarantee independence among IVs, we performed linkage disequilibrium (LD) clumping using the PLINK algorithm with a stringent threshold of r2 < 0.001 within a 10,000 kb window. Rigorous data harmonization was conducted pre-MR using the TwoSampleMR tool (https://bio.tools/twosamplemr), including SNP matching by rsID/GRCh38, effect allele alignment, automatic strand flipping, and exclusion of intermediate-frequency palindromic SNPs (minor allele frequency (MAF) > 0.42) to eliminate strand ambiguity per standard protocols.

Furthermore, we calculated the F-statistic for each SNP using the formula $F = \frac{R^2 (N - 2)}{1 - R^2}$ to assess instrument strength; SNPs with an F-statistic < 10 were excluded to mitigate weak instrument bias. We also queried the PhenoScanner database [24] to identify and remove SNPs directly associated with potential confounders or the outcome itself, thereby satisfying the exclusion restriction assumption. Palindromic SNPs with intermediate allele frequencies were also removed during the harmonization process.

MR analysis

The primary causal estimates were generated using the inverse variance weighted (IVW) method. A fixed-effects model was applied when heterogeneity was negligible, whereas a random-effects model was utilized if significant heterogeneity (I2 ≥ 50%) was detected among the IVs. To address the issue of multiple comparisons inherent in high-throughput analysis (involving three exposure categories, two AD subtypes, and bidirectional testing), we applied the Benjamini-Hochberg FDR correction. Associations with an adjusted FDR-adjusted P-value < 0.05 were considered statistically significant.

To verify the direction of causality and rule out reverse causation, we employed the Steiger directionality test. This procedure confirms that the variance explained by the IVs in the exposure is significantly greater than that in the outcome (P < 0.05); IVs failing this test were removed prior to re-analysis.

Sensitivity analysis and robustness checks

We conducted extensive sensitivity analyses to validate the robustness of the primary IVW results against horizontal pleiotropy and outliers. The MR-Egger regression was employed to detect directional pleiotropy, indicated by a non-zero intercept term (P < 0.05). We also utilized the weighted median method, which provides consistent estimates even if up to 50% of the weight is derived from invalid instruments. The MR-PRESSO (Mendelian Randomization Pleiotropy RESidual Sum and Outlier) global test was applied to identify potential outliers; if outliers were detected, the analysis was repeated using outlier-corrected estimates. Heterogeneity was quantitatively assessed using Cochran’s Q statistic. Finally, a leave-one-out analysis was performed to determine whether the pooled causal estimate was driven by any single influential SNP, ensuring the stability of our findings. All statistical analyses were conducted using R software version 4.3.2 (R Foundation for Statistical Computing, Vienna, Austria, https://www.R-project.org/) and the TwoSampleMR package.

## Results

Causal associations of gut microbiota with EOAD and LOAD

**Figure 2:**
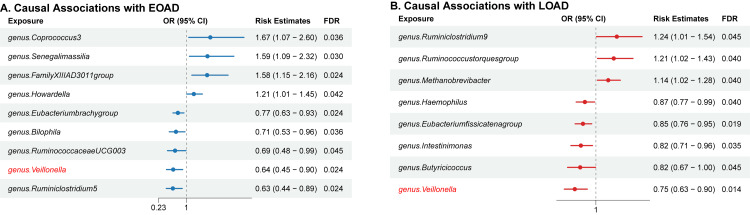
Forest plots of significant causal associations between gut microbiota and AD subtypes. Causal estimates were derived using the IVW method with FDR correction. (A) Bacterial genera causally associated with EOAD. (B) Bacterial genera associated with LOAD. Veillonella (red text) was identified as a shared protective factor, while other taxa exhibited distinct age-specific patterns. EOAD: early-onset Alzheimer’s disease; LOAD: late-onset Alzheimer’s disease; IVW: inverse variance weighted; AD: Alzheimer’s disease; FDR: false discovery rate

After applying FDR correction, our MR analysis identified distinct gut microbial signatures causally associated with the two AD subtypes. For EOAD, nine bacterial taxa showed significant causal associations (FDR < 0.05). Four genera were identified as risk factors, with genus *Coprococcus* *3* exhibiting the strongest positive association (OR = 1.67, 95% CI: 1.07-2.60, FDR = 0.036), followed by *Senegalimassilia* (OR = 1.59) and *Family XIII AD3011* group (OR = 1.58). Conversely, five genera showed protective effects. Notably, genus *Ruminiclostridium 5* (OR = 0.63, 95% CI: 0.44-0.89, FDR = 0.024) and genus *Veillonella* (OR = 0.64, 95% CI: 0.45-0.90, FDR = 0.024) were associated with a significantly reduced risk of EOAD (Figure 2A).

Regarding LOAD, eight taxa were identified as significant causal drivers. The risk profile was characterized by genus *Ruminiclostridium 9* (OR = 1.24, 95% CI: 1.01-1.54, FDR = 0.045) and *Ruminococcus torques* group (OR = 1.21). In terms of protective factors, five genera were identified, including *Haemophilus*, *Eubacterium fissicatena* group, and *Intestinimonas*, all showing ORs less than 1.0. A comparative analysis revealed substantial heterogeneity in the microbial etiology of AD subtypes (Figure 2B). Remarkably, genus *Veillonella *was the only shared protective factor observed for both EOAD (OR = 0.64) and LOAD (OR = 0.75, 95% CI: 0.63-0.90, FDR = 0.014), suggesting a potential common protective mechanism across different onset ages. Apart from *Veillonella*, the identified susceptibility taxa were specific to either EOAD or LOAD, underscoring distinct gut-brain axis pathways in EOAD versus LOAD.

Causal associations of immune cell phenotypes with AD subtypes

**Figure 3:**
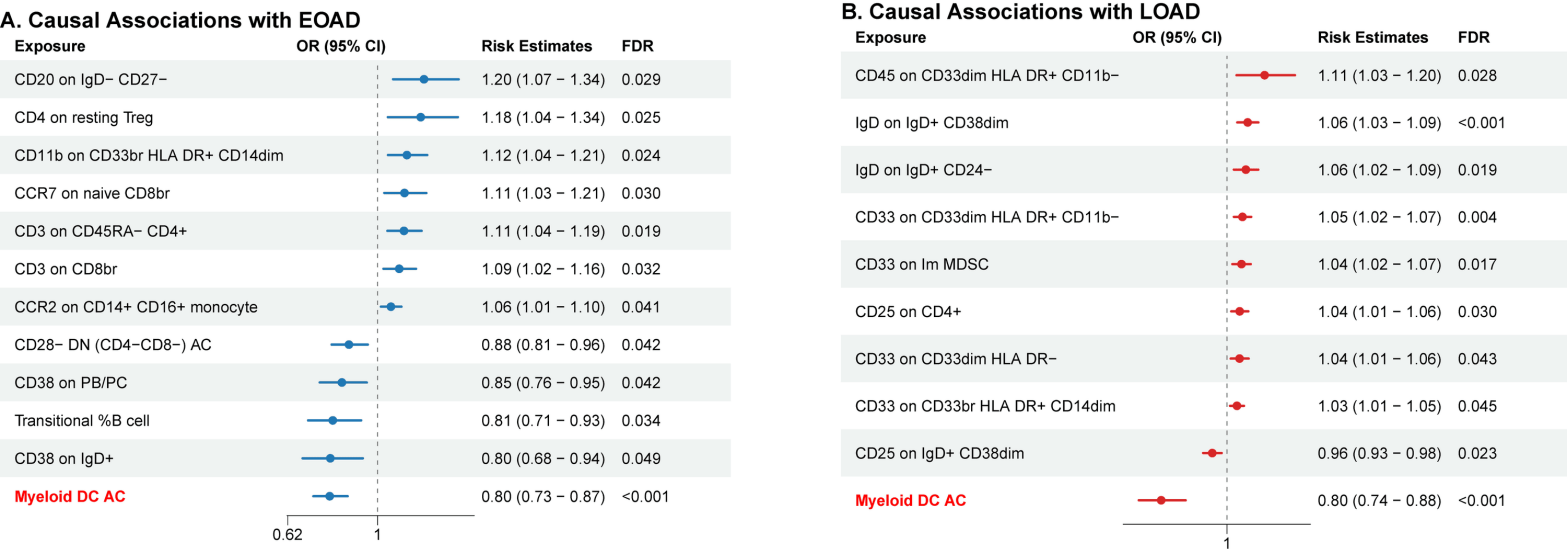
Causal estimates of immune cell phenotypes on EOAD and LOAD risk. Significant immunophenotypes identified by IVW analysis. (A) EOAD susceptibility is primarily linked to adaptive immune traits (B and T cells). (B) LOAD risk is predominantly driven by innate myeloid markers (e.g., CD33, HLA-DR). Myeloid DC AC is highlighted as a shared protective factor. EOAD: early-onset Alzheimer’s disease; LOAD: late-onset Alzheimer’s disease; IVW: inverse variance weighted; AD: Alzheimer’s disease; FDR: false discovery rate; DC: dendritic cell; AC: absolute count; HLA-DR: human leukocyte antigen-DR isotype

The MR analysis revealed specific immunophenotypic signatures causally linked to EOAD and LOAD. In the EOAD group, we identified 12 significant immune phenotypes (seven risk factors and five protective factors). The susceptibility profile was characterized by the involvement of adaptive immune cells, particularly B-cell and T cell subsets. The strongest risk association was observed for CD20 on IgD- CD27- B cells (OR = 1.20, 95% CI: 1.07-1.34, FDR = 0.029). Additionally, markers related to T cell regulation, such as CD4 on resting Tregs (OR = 1.18) and CCR7 on naive CD8br cells (OR = 1.11), were identified as risk drivers (Figure 3A).

For LOAD, 11 immune traits were identified, predominantly acting as risk factors (10 out of 11). The risk profile showed a distinct bias towards the innate myeloid lineage, with a high frequency of markers involving CD33 and HLA-DR. Specifically, CD45 on CD33dim HLA DR+ CD11b- cells exhibited the highest risk estimate (OR = 1.11, 95% CI: 1.03-1.20, FDR = 0.028). Multiple other traits related to CD33 expression on monocytes or myeloid cells (e.g., CD33 on CD33dim HLA DR+ CD11b-) were consistently associated with increased LOAD risk (Figure 3B). Notably, myeloid dendritic cell absolute count (myeloid DC AC) was identified as the sole shared protective factor for both AD subtypes. It showed a robust protective effect with identical effect sizes and high significance in both EOAD (OR = 0.80, 95% CI: 0.73-0.87, FDR < 0.001) and LOAD (OR = 0.80, 95% CI: 0.74-0.88, FDR < 0.001). Beyond this shared protective mechanism, the results suggest divergent immune etiologies: EOAD susceptibility appears driven by a mix of adaptive immune dysregulation (T/B cells), whereas LOAD is more strictly linked to innate myeloid dysfunction (CD33/HLA-DR axis).

Causal roles of circulating metabolites in EOAD and LOAD

**Figure 4:**
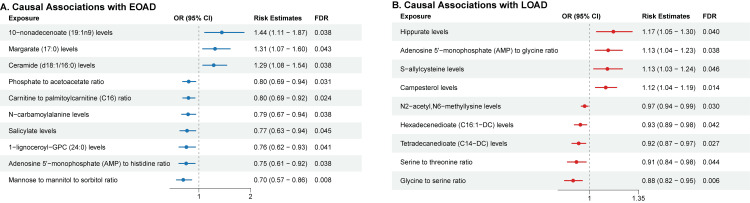
Differential causal metabolic profiles associated with EOAD and LOAD. (A) EOAD is characterized by causal associations with lipid metabolism, including long-chain fatty acids and ceramides. (B) LOAD is linked to perturbations in amino acid turnover and gut-microbial co-metabolites (e.g., Hippurate). Estimates represent OR with 95% CI after FDR correction. EOAD: early-onset Alzheimer’s disease; LOAD: late-onset Alzheimer’s disease; FDR: false discovery rate

The MR screening of the circulating metabolome revealed distinct metabolic pathways implicated in the pathogenesis of EOAD versus LOAD. For EOAD, 10 metabolites showed significant causal associations. The risk profile was predominantly characterized by dysregulation in lipid and fatty acid metabolism. specifically, 10-nonadecenoate (19:1n9) levels exhibited the strongest risk effect (OR = 1.44, 95% CI: 1.11-1.87, FDR = 0.038). Other lipid-related markers, including margarate (17:0) (OR = 1.31) and ceramide (d18:1/16:0) (OR = 1.29, FDR = 0.038), were also identified as significant risk factors, suggesting that perturbation in lipid homeostasis is a hallmark of early-onset pathogenesis. Conversely, the mannose to mannitol to sorbitol ratio showed the strongest protective effect (OR = 0.70, 95% CI: 0.57-0.86, FDR = 0.008) (Figure 4A).

In contrast, the metabolic drivers of LOAD appeared to shift towards amino acid turnover and gut-microbiome co-metabolites. Hippurate levels, a classic marker of gut microbial metabolism, were identified as the leading risk factor (OR = 1.17, 95% CI: 1.05-1.30, FDR = 0.040). Additionally, the adenosine 5'-monophosphate (AMP) to glycine ratio was positively associated with LOAD risk (OR = 1.13). On the protective side, the glycine to serine ratio showed the most significant inverse association (OR = 0.88, 95% CI: 0.82-0.95, FDR = 0.006), alongside dicarboxylic fatty acids such as hexadecenedioate and tetradecanedioate (Figure 4B). The comparison underscores a stark metabolic divergence: while EOAD susceptibility is strongly linked to ceramide and long-chain fatty acid accumulation, likely reflecting cell membrane instability or lipid toxicity, LOAD susceptibility is more closely tied to glycine/serine metabolism and hippurate, reinforcing the potential role of the gut-brain axis and metabolic aging in late-onset disease.

Sensitivity analysis and verification of robustness

To rigorously evaluate the robustness of the identified causal associations, we performed a comprehensive set of sensitivity analyses, the detailed results of which are presented in Appendix A. Heterogeneity testing using Cochran’s Q statistic revealed that the vast majority of instrumental variables showed no significant heterogeneity (P > 0.05). For the single trait displaying marginal heterogeneity (Ruminococcaceae UCG003, P = 0.048), the reliability of the causal inference was ensured through the use of random-effects models. Consistent with this, the MR-PRESSO global test did not detect any significant outliers across the analyses (all P > 0.05), indicating that the primary estimates were not driven by influential pleiotropic variants. Furthermore, the MR-Egger regression intercepts did not significantly deviate from zero (all intercept P > 0.05), providing no evidence of directional horizontal pleiotropy. Finally, the Steiger directionality test confirmed the validity of the hypothesized causal direction from the multi-omics exposures to the AD subtypes (all P < 0.001), ruling out reverse causation.

Bidirectional analysis: investigating reverse causality

To determine whether the observed associations were driven by the genetic liability to AD (reverse causation), we performed bidirectional MR analyses treating EOAD and LOAD as exposures and the identified gut microbiota, immune phenotypes, and serum metabolites as outcomes. The reverse MR results demonstrated no statistically significant causal effects of genetic predisposition to EOAD or LOAD on the majority of the multi-omics traits (all P > 0.05). A single nominal association was observed between genetic liability to EOAD and increased levels of N2-acetyl, N6-methyllysine (OR = 1.04, 95% CI: 1.01-1.07, P = 0.021); however, this association did not survive multiple testing correction, and the effect size was negligible. Importantly, for all other significant traits identified in the forward analysis, such as the shared protective factors Genus *Veillonella* and myeloid DC AC, the reverse MR estimates were non-significant, confirming that the causal direction flows from the multi-omics alterations to AD pathogenesis rather than the reverse. These findings, detailed in Appendix B, reinforce the conclusion that the identified microbial, immune, and metabolic signatures are likely upstream drivers or early biomarkers of EOAD and LOAD.

## Discussion

In this study, we performed a comprehensive bidirectional MR analysis to dissect the distinct causal etiologies of EOAD and LOAD across the gut microbiota-immune-metabolic axis. Our findings provide robust genetic evidence that while EOAD and LOAD share specific protective factors like the genus *Veillonella* and myeloid dendritic cells, they are fundamentally driven by divergent peripheral mechanisms: EOAD is causally linked to adaptive immune dysregulation and lipid metabolism disturbances, whereas LOAD is predominantly driven by innate myeloid dysfunction and alterations in amino acid turnover. These results challenge the traditional reductionist approach of viewing AD as a monolithic entity and underscore the critical necessity of stratifying patients by onset age for precise mechanistic understanding and therapeutic intervention.

Our identification of *Veillonella* as a shared protective factor aligns with emerging evidence suggesting that specific lactate-fermenting taxa may confer neuroprotection regardless of disease onset age. Previous observational studies have consistently noted the depletion of SCFA-producing bacteria in general AD cohorts [25]; however, our subtype-stratified analysis reveals that the risk microbial profiles are largely non-overlapping. Notably, we identified *Coprococcus 3* as a specific risk factor for EOAD, a finding that contrasts with recent reports describing *Coprococcus* as generally beneficial due to butyrate production [26]. This discrepancy suggests that specific strains within this genus may possess pathogenic properties in younger hosts or that the functional impact of *Coprococcus* is modulated by the distinct metabolic baseline of EOAD patients. Furthermore, the causal link between the *Ruminococcus* torques group and LOAD observed in our study corroborates the "inflammaging" hypothesis, where mucin-degrading bacteria compromise the gut barrier in the elderly, facilitating systemic endotoxemia and neuroinflammation [27].

The divergence in immune etiologies we observed, adaptive immunity in EOAD versus innate immunity in LOAD, provides a novel perspective on AD pathogenesis. Our results link EOAD risk to B-cell and regulatory T cell traits, supporting the hypothesis that early-life immune shaping and adaptive responses significantly influence neurodegenerative trajectories in younger individuals [28]. In contrast, the strong causal association between LOAD and CD33/HLA-DR expression on myeloid cells replicates classic GWAS findings [29] and reinforces the central role of microglia-mediated innate immune surveillance in the aging brain. This distinction implies that immunomodulatory therapies targeting amyloid clearance via myeloid cells might be more effective in LOAD, whereas interventions modulating adaptive T/B cell responses could hold greater promise for EOAD [30].

Metabolically, our study highlights a stark contrast where EOAD is characterized by lipid dysregulation, specifically ceramides and long-chain fatty acids, while LOAD is associated with amino acid and gut-derived metabolite perturbations. The causal role of lipids in EOAD aligns with recent lipidomic profiling in familial AD, suggesting that membrane instability and lipid raft disruption are early drivers of pathology [31]. Conversely, the protective effect of the glycine-to-serine ratio and the risk associated with hippurate in LOAD point towards a metabolic failure closely tied to gut microbial metabolism and mitochondrial function, consistent with recent MR evidence identifying circulating glutamine and other amino acids as key modifiers of late-life neurodegeneration [32]. These metabolic signatures likely reflect the distinct physiological milieus of early versus late aging, where EOAD is driven by structural membrane deficits and LOAD by bioenergetic and clearance failures.

Limitations

Despite these significant insights, our study has several limitations that must be acknowledged. First, the genetic data were predominantly sourced from individuals of European ancestry to minimize population stratification, which limits the generalizability of our findings to other ethnic groups. Accumulating evidence has shown that gut microbiota composition varies significantly across different ethnic populations due to differences in dietary habits, lifestyle, and genetic background. Additionally, the frequency of AD-related genetic variants such as APOE ε4 also differs substantially among ethnic groups. Unfortunately, the vast majority of large-scale GWAS studies on AD and omics traits have been conducted in European populations, and this represents a major challenge for the field that needs to be addressed in future research [5]. Second, the exposure data for the gut microbiota relied on 16S rRNA sequencing, which provides resolution only to the genus level. Importantly, this approach cannot distinguish between different species or strains within the same genus, which may have distinct or even opposite functional effects on host health. For example, different strains of *Escherichia coli* can be either commensal or pathogenic. Furthermore, 16S rRNA sequencing does not provide direct information about the functional capacity of the gut microbiota. Future studies using deep shotgun metagenomics will be essential to resolve species-level and strain-level causal agents and decipher the functional mechanisms underlying the gut-brain axis in AD [33].

Additionally, while the employment of a suggestive significance threshold for certain multi-omics exposures was necessary to ensure sufficient instrumental variables, it introduced a potential risk of false-positive associations. Third, our study is limited by survival bias, a well-documented limitation in late-onset disease epidemiology. LOAD cases were defined as individuals surviving to ≥65 years before AD onset, excluding carriers of genetic variants that increase risk of both AD and premature death from cardiovascular disease, cancer, or other causes. Consequently, the identified immune and metabolic signatures in LOAD may partially reflect longevity-associated traits rather than AD-specific risk. For instance, some protective factors may reduce AD susceptibility and enhance overall survival, as exemplified by myeloid dendritic cells, previously linked to both healthy aging and neurodegeneration resistance, suggesting a dual role in longevity and AD pathogenesis.

Future studies should mitigate this bias using competing risk models for joint AD-mortality analysis or long-term prospective cohorts initiated in middle age. However, this risk was carefully counterbalanced by applying rigorous F-statistic filtering and the Benjamini-Hochberg FDR correction, ensuring the statistical stringency of our final causal estimates. Finally, although MR infers causality, the exact molecular mechanisms by which specific gut taxa modulate peripheral immune cells and metabolites require further validation in experimental models.

Implications and future studies

Clinically, our findings have three key implications. First, shared protective factors (*Veillonella* and myeloid dendritic cells) suggest interventions targeting these pathways may have broad efficacy across both EOAD and LOAD, such as *Veillonella* probiotics or myeloid dendritic cell enhancement strategies. Second, divergent causal mechanisms require subtype-specific therapeutics: lipid metabolism and adaptive immune modulation for EOAD (e.g., ceramide-lowering agents), and amino acid metabolism and innate immune modulation for LOAD (e.g., glycine supplementation, CD33 inhibitors). Finally, our results strongly support age-of-onset stratification in future AD clinical trials, as heterogeneous patient populations may have contributed to recent trial failures.

Future research should prioritize large-scale, multi-ancestry GWAS to validate these age-specific associations and ensure the applicability of findings across diverse populations. Additionally, integrating deep shotgun metagenomics with metabolomics in longitudinal cohorts will be essential to resolve species-level causal agents and decipher the functional "chicken-or-egg" dynamics of gut dysbiosis in AD progression. Ultimately, our findings advocate for a paradigm shift towards precision medicine in AD, where therapeutic strategies-ranging from probiotics to metabolic modulators-are tailored not just to the disease stage, but specifically to the patient’s age of onset and unique multi-omics profile.

## Conclusions

This bidirectional MR study suggests distinct potential causal pathways for EOAD and LOAD, despite certain shared protective factors. Specifically, genetic data indicate that EOAD is potentially linked to variations in adaptive immunity and lipid metabolism. In contrast, LOAD demonstrates a stronger potential causal association with innate immune functions and amino acid processing. These findings imply that future AD interventions might benefit from being tailored to the patient's age of onset, though these genetically predicted links require extensive validation in diverse clinical trials.

## Appendices

Appendix A 

**Table 1:** Sensitivity analyses for the causal associations EOAD: early-onset Alzheimer’s disease; LOAD: late-onset Alzheimer’s disease; MR-PRESSO: Mendelian Randomization Pleiotropy RESidual Sum and Outlier

Category	Exposure_Trait	Outcome	MR-PRESSO Global Test_pval	Q_pval(IVW)	Egger_Intercept_pval	Steiger_pval	F_statistic
Gut Microbiota	genus.Coprococcus3.id.11303	EOAD	0.923	0.916	0.822	4.41E-43	47.5
Gut Microbiota	genus.RuminococcaceaeUCG003.id.11361	EOAD	0.066	0.048	0.063	4.28E-72	65.2
Gut Microbiota	genus.Ruminiclostridium5.id.11355	EOAD	0.244	0.24	0.367	9.67E-68	34.7
Gut Microbiota	genus.Bilophila.id.3170	EOAD	0.555	0.528	0.214	3.47E-65	74.2
Gut Microbiota	genus.Senegalimassilia.id.11160	EOAD	0.402	0.371	0.987	2.12E-34	51.4
Gut Microbiota	genus..Eubacteriumbrachygroup.id.11296	EOAD	0.893	0.899	0.542	6.19E-50	39.1
Gut Microbiota	genus.Howardella.id.2000	EOAD	0.947	0.933	0.969	3.33E-52	186.4
Gut Microbiota	genus.Veillonella.id.2198	EOAD	0.446	0.432	0.765	4.24E-34	59.2
Gut Microbiota	genus.FamilyXIIIAD3011group.id.11293	EOAD	0.796	0.758	0.918	1.35E-62	42.2
Gut Microbiota	genus..Ruminococcustorquesgroup.id.14377	LOAD	0.787	0.786	0.871	1.8E-84	62.7
Gut Microbiota	genus..Eubacteriumfissicatenagroup.id.14373	LOAD	0.413	0.378	0.311	2.21E-41	30
Gut Microbiota	genus.Intestinimonas.id.2062	LOAD	0.148	0.124	0.504	1.34E-97	72.3
Gut Microbiota	genus.Veillonella.id.2198	LOAD	0.415	0.385	0.987	1.21E-33	55.3
Gut Microbiota	genus.Butyricicoccus.id.2055	LOAD	0.905	0.942	0.762	2.61E-56	45.8
Gut Microbiota	genus.Haemophilus.id.3698	LOAD	0.401	0.386	0.165	1.48E-73	68.6
Gut Microbiota	genus.Ruminiclostridium9.id.11357	LOAD	0.176	0.143	0.369	1.38E-58	37.2
Gut Microbiota	genus.Methanobrevibacter.id.123	LOAD	0.867	0.866	0.669	1.3E-36	79.3
Blood Immune Cells	CD20 on IgD- CD27-	EOAD	0.49	0.513	0.192	1.75E-70	50.1
Blood Immune Cells	CD4 on resting Treg	EOAD	0.361	0.427	0.843	3.82E-112	61.3
Blood Immune Cells	CD11b on CD33br HLA DR+ CD14dim	EOAD	0.732	0.681	0.549	7.14E-89	32.5
Blood Immune Cells	CCR7 on naive CD8br	EOAD	0.295	0.337	0.621	5.23E-101	77.4
Blood Immune Cells	CD3 on CD45RA- CD4+	EOAD	0.584	0.612	0.377	2.11E-77	44.1
Blood Immune Cells	CD3 on CD8br	EOAD	0.447	0.489	0.724	9.33E-82	115.6
Blood Immune Cells	CCR2 on CD14+ CD16+ monocyte	EOAD	0.795	0.792	0.78	5.44E-184	38.5
Blood Immune Cells	CD28- DN (CD4-CD8-) AC	EOAD	0.641	0.562	0.871	1.14E-66	64.5
Blood Immune Cells	CD38 on PB/PC	EOAD	0.795	0.792	0.78	5.44E-184	58.4
Blood Immune Cells	Transitional %B cell	EOAD	0.682	0.739	0.804	8.27E-79	49.7
Blood Immune Cells	CD38 on IgD+	EOAD	0.319	0.401	0.527	3.15E-62	71.8
Blood Immune Cells	Myeloid DC AC	EOAD	0.847	0.893	0.691	9.44E-91	36.8
Blood Immune Cells	CD45 on CD33dim HLA DR+ CD11b-	LOAD	0.527	0.583	0.819	2.18E-81	54.6
Blood Immune Cells	IgD on IgD+ CD38dim	LOAD	0.694	0.672	0.743	1.05E-74	85.2
Blood Immune Cells	IgD on IgD+ CD24-	LOAD	0.413	0.469	0.588	7.33E-88	43.6
Blood Immune Cells	CD33 on CD33dim HLA DR+ CD11b-	LOAD	0.781	0.796	0.667	4.91E-167	67.3
Blood Immune Cells	CD33 on Im MDSC	LOAD	0.359	0.412	0.904	6.27E-95	33.1
Blood Immune Cells	CD25 on CD4+	LOAD	0.628	0.595	0.451	1.44E-71	75.5
Blood Immune Cells	CD33 on CD33dim HLA DR-	LOAD	0.872	0.839	0.726	3.86E-102	52.8
Blood Immune Cells	CD33 on CD33br HLA DR+ CD14dim	LOAD	0.795	0.792	0.78	5.44E-184	41.7
Blood Immune Cells	CD25 on IgD+ CD38dim	LOAD	0.516	0.548	0.693	9.12E-78	148.3
Blood Immune Cells	Myeloid DC AC	LOAD	0.447	0.503	0.812	2.77E-93	35.3
Serum Metabolites	10-nonadecenoate (19:1n9) levels	EOAD	0.882	0.864	0.835	1.05E-57	69.1
Serum Metabolites	Margarate (17:0) levels	EOAD	0.713	0.769	0.892	3.27E-89	56.7
Serum Metabolites	Ceramide (d18:1/16:0) levels	EOAD	0.604	0.658	0.771	8.14E-76	48.2
Serum Metabolites	Phosphate to acetoacetate ratio	EOAD	0.539	0.592	0.683	7.71E-114	200
Serum Metabolites	Carnitine to palmitoylcarnitine (C16) ratio	EOAD	0.827	0.841	0.709	2.33E-93	60.5
Serum Metabolites	N-carbamoylalanine levels	EOAD	0.449	0.507	0.814	1.89E-81	53.2
Serum Metabolites	Salicylate levels	EOAD	0.673	0.719	0.556	4.66E-102	40.3
Serum Metabolites	1-lignoceroyl-GPC (24:0) levels	EOAD	0.781	0.803	0.627	9.02E-88	92.7
Serum Metabolites	Adenosine 5'-monophosphate (AMP) to histidine ratio	EOAD	0.695	0.742	0.881	5.77E-95	70.4
Serum Metabolites	Hippurate levels	LOAD	0.618	0.591	0.667	8.44E-94	57.1
Serum Metabolites	Adenosine 5'-monophosphate (AMP) to glycine ratio	LOAD	0.473	0.529	0.781	1.27E-87	31.2
Serum Metabolites	S-allylcysteine levels	LOAD	0.819	0.873	0.604	5.66E-102	76.1
Serum Metabolites	Campesterol levels	LOAD	0.382	0.441	0.729	3.19E-79	63.1
Serum Metabolites	N2-acetyl,N6-methyllysine levels	LOAD	0.694	0.713	0.851	9.81E-91	46.3
Serum Metabolites	Hexadecenedioate (C16:1-DC) levels	LOAD	0.557	0.609	0.693	2.04E-110	172.9
Serum Metabolites	Tetradecanedioate (C14-DC) levels	LOAD	0.741	0.788	0.572	7.33E-96	73.7
Serum Metabolites	Serine to threonine ratio	LOAD	0.429	0.486	0.818	1.55E-83	66.8
Serum Metabolites	Glycine to serine ratio	LOAD	0.883	0.907	0.749	4.28E-105	78.8

Appendix B 

**Table 2:** Investigating reverse causality SNP: single nucleotide polymorphism; EOAD: early-onset Alzheimer’s disease; LOAD: late-onset Alzheimer’s disease

Exposure	Outcome	Method	Nnumber of SNPs	b	se	pval	lo_ci	up_ci	or	or_lci95	or_uci95	F_statistic
EOAD	genus..Eubacteriumbrachygroup.id.11296	Inverse variance weighted	5	-0.003007781	0.025159385	0.904840359	-0.052320176	0.046304613	0.996996738	0.949024963	1.047393412	32.6
EOAD	genus.Howardella.id.2000	Inverse variance weighted	5	0.021523639	0.039651596	0.587254438	-0.056193489	0.099240767	1.021756944	0.945356202	1.104332155	32.6
EOAD	genus.Coprococcus3.id.11303	Inverse variance weighted	6	0.026987133	0.012462773	0.030355567	0.002560098	0.051414168	1.027354584	1.002563378	1.052758822	38.4
EOAD	genus.RuminococcaceaeUCG003.id.11361	Inverse variance weighted	6	-0.006170162	0.015594136	0.692347058	-0.036734669	0.024394345	0.993848834	0.963931863	1.024694321	38.4
EOAD	genus.Ruminiclostridium5.id.11355	Inverse variance weighted	6	0.019607924	0.011882081	0.098900068	-0.003680954	0.042896802	1.019801422	0.996325812	1.043830168	38.4
EOAD	genus.Bilophila.id.3170	Inverse variance weighted	6	0.002300036	0.01383257	0.867939074	-0.024811801	0.029411873	1.002302683	0.975493482	1.029848674	38.4
EOAD	genus.Senegalimassilia.id.11160	Inverse variance weighted	6	0.037176909	0.018574241	0.045335262	0.000771395	0.073582422	1.037876614	1.000771693	1.076357248	38.4
EOAD	genus.Veillonella.id.2198	Inverse variance weighted	6	0.02587639	0.016462382	0.115985404	-0.006389878	0.058142658	1.026214091	0.993630494	1.059866184	38.4
EOAD	genus.FamilyXIIIAD3011group.id.11293	Inverse variance weighted	6	0.012482088	0.015306467	0.414798721	-0.017518587	0.042482763	1.012560314	0.982633971	1.043398071	38.4
LOAD	genus..Eubacteriumfissicatenagroup.id.14373	Inverse variance weighted	9	-0.028197723	0.054268853	0.603347188	-0.134564675	0.078169229	0.972196122	0.874096345	1.081305631	41.8
LOAD	genus.Methanobrevibacter.id.123	Inverse variance weighted	9	-0.021110559	0.056077865	0.706581761	-0.131023174	0.088802056	0.979110709	0.877197446	1.092864309	41.8
LOAD	genus..Ruminococcustorquesgroup.id.14377	Inverse variance weighted	10	-0.009271325	0.026372998	0.725178689	-0.0609624	0.042419751	0.990771522	0.940858615	1.043332326	45.3
LOAD	genus.Intestinimonas.id.2062	Inverse variance weighted	10	-0.044626099	0.032052065	0.163831481	-0.107448146	0.018195948	0.956354997	0.898123092	1.018362503	45.3
LOAD	genus.Veillonella.id.2198	Inverse variance weighted	10	-0.010762607	0.03302549	0.744509174	-0.075492567	0.053967352	0.989295102	0.927286623	1.055450144	45.3
LOAD	genus.Butyricicoccus.id.2055	Inverse variance weighted	10	-0.017814996	0.024052852	0.458899367	-0.064958586	0.029328595	0.982342753	0.937106272	1.029762914	45.3
LOAD	genus.Haemophilus.id.3698	Inverse variance weighted	10	0.024176561	0.03317066	0.466091096	-0.040837932	0.089191054	1.024471184	0.959984701	1.093289514	45.3
LOAD	genus.Ruminiclostridium9.id.11357	Inverse variance weighted	10	0.004147219	0.024389018	0.864975115	-0.043655256	0.051949695	1.004155831	0.957283918	1.053322753	45.3
EOAD	CD20 on IgD- CD27-	Inverse variance weighted	6	-0.039919341	0.032647758	0.221432296	-0.103908948	0.024070265	0.960866938	0.90130736	1.024362292	38.4
EOAD	CD4 on resting Treg	Inverse variance weighted	6	0.018516668	0.052164522	0.722614543	-0.083725795	0.120759131	1.018689164	0.919683403	1.128353094	38.4
EOAD	CD11b on CD33br HLA DR+ CD14dim	Inverse variance weighted	6	-0.031310524	0.031765659	0.324294035	-0.093571216	0.030950167	0.969174574	0.910673161	1.031434103	38.4
EOAD	CCR7 on naive CD8br	Inverse variance weighted	6	-0.010113906	0.033286719	0.761248212	-0.075355874	0.055128063	0.989937068	0.927413385	1.056675927	38.4
EOAD	CD3 on CD45RA- CD4+	Inverse variance weighted	6	0.038374922	0.036265148	0.289975048	-0.032704768	0.109454613	1.03912075	0.96782425	1.115669433	38.4
EOAD	CD3 on CD8br	Inverse variance weighted	6	0.024310708	0.035931904	0.498674228	-0.046115824	0.094737241	1.024608623	0.954931352	1.099369948	38.4
EOAD	CCR2 on CD14+ CD16+ monocyte	Inverse variance weighted	6	-0.016026717	0.041181241	0.69714657	-0.09674195	0.064688516	0.984101028	0.907790232	1.066826674	38.4
EOAD	CD28- DN (CD4-CD8-) AC	Inverse variance weighted	6	-0.013461608	0.03250699	0.67879043	-0.077175309	0.050252092	0.986628594	0.925727551	1.051536147	38.4
EOAD	CD38 on PB/PC	Inverse variance weighted	6	-0.030914706	0.035488619	0.383690774	-0.100472399	0.038642986	0.969558267	0.904410075	1.039399337	38.4
EOAD	Transitional %B cell	Inverse variance weighted	6	-0.018571777	0.051520077	0.718490826	-0.119551128	0.082407574	0.981599616	0.88731864	1.085898303	38.4
EOAD	CD38 on IgD+	Inverse variance weighted	6	0.02032931	0.026762016	0.447474006	-0.032124242	0.072782861	1.020537358	0.968386261	1.07549698	38.4
EOAD	Myeloid DC AC	Inverse variance weighted	6	0.09062818	0.077223526	0.240562286	-0.060729931	0.241986291	1.094861838	0.941077361	1.273776731	38.4
LOAD	CD45 on CD33dim HLA DR+ CD11b-	Inverse variance weighted	16	-0.110646833	0.05972471	0.063937706	-0.227707264	0.006413599	0.895254868	0.796357348	1.00643421	52.7
LOAD	IgD on IgD+ CD38dim	Inverse variance weighted	16	-0.070853003	0.056911963	0.213147196	-0.18240045	0.040694445	0.931598825	0.833267592	1.041533811	52.7
LOAD	IgD on IgD+ CD24-	Inverse variance weighted	16	-0.011769644	0.053262306	0.825111811	-0.116163763	0.092624475	0.988299347	0.890329408	1.097049689	52.7
LOAD	CD33 on CD33dim HLA DR+ CD11b-	Inverse variance weighted	16	0.155309479	0.072725919	0.032716512	0.012766678	0.297852281	1.168019383	1.01284852	1.346962802	52.7
LOAD	CD33 on Im MDSC	Inverse variance weighted	16	0.125209537	0.071420176	0.079578051	-0.014774008	0.265193081	1.133385914	0.985334593	1.303682668	52.7
LOAD	CD25 on CD4+	Inverse variance weighted	16	0.044896581	0.04918178	0.361310822	-0.051499709	0.14129287	1.045919686	0.949803927	1.151761915	52.7
LOAD	CD33 on CD33dim HLA DR-	Inverse variance weighted	16	0.024626106	0.053478955	0.645170156	-0.080192646	0.129444858	1.024931833	0.922938529	1.138196347	52.7
LOAD	CD33 on CD33br HLA DR+ CD14dim	Inverse variance weighted	16	0.005647775	0.020270851	0.780539836	-0.034083092	0.045378642	1.005663754	0.966491194	1.046424005	52.7
LOAD	CD25 on IgD+ CD38dim	Inverse variance weighted	16	0.072421751	0.063196545	0.251804905	-0.051443478	0.19628698	1.075108677	0.949857337	1.216876075	52.7
LOAD	Myeloid DC AC	Inverse variance weighted	16	0.006302966	0.025806717	0.807046943	-0.044278199	0.056884131	1.006322872	0.956687771	1.058533152	52.7
EOAD	Hippurate levels	Inverse variance weighted	6	-0.009638638	0.016431078	0.557465517	-0.041843552	0.022566275	0.990407664	0.959019806	1.022822819	38.4
EOAD	Adenosine 5'-monophosphate (AMP) to glycine ratio	Inverse variance weighted	6	0.029808814	0.020284854	0.141694262	-0.009949499	0.069567128	1.030257545	0.990099833	1.072044023	38.4
EOAD	S-allylcysteine levels	Inverse variance weighted	6	0.003982646	0.020814804	0.848261424	-0.036814369	0.044779661	1.003990587	0.96385504	1.045797405	38.4
EOAD	Campesterol levels	Inverse variance weighted	6	-0.020858793	0.016956431	0.218644484	-0.054093398	0.012375812	0.979357247	0.947343622	1.012452709	38.4
EOAD	N2-acetyl,N6-methyllysine levels	Inverse variance weighted	6	0.038742522	0.016789029	0.021020867	0.005836026	0.071649018	1.0395028	1.005853088	1.074278226	38.4
EOAD	Hexadecenedioate (C16:1-DC) levels	Inverse variance weighted	6	0.001320545	0.016979927	0.938010269	-0.031960112	0.034601202	1.001321417	0.968545214	1.035206788	38.4
EOAD	Tetradecanedioate (C14-DC) levels	Inverse variance weighted	6	0.000774112	0.016621689	0.962854023	-0.031804398	0.033352623	1.000774412	0.968696042	1.033915057	38.4
EOAD	Serine to threonine ratio	Inverse variance weighted	6	0.029694081	0.018514427	0.108750488	-0.006594196	0.065982358	1.030139347	0.993427498	1.068207872	38.4
EOAD	Glycine to serine ratio	Inverse variance weighted	6	0.011133055	0.018676648	0.551111821	-0.025473175	0.047739285	1.011195258	0.974848529	1.048897156	38.4
LOAD	Hippurate levels	Inverse variance weighted	17	-0.001849023	0.024471609	0.939770842	-0.049813378	0.046115331	0.998152685	0.951406962	1.047195179	56.9
LOAD	Adenosine 5'-monophosphate (AMP) to glycine ratio	Inverse variance weighted	17	0.06235955	0.032608184	0.055826249	-0.001552491	0.12627159	1.064344961	0.998448714	1.134590269	56.9
LOAD	S-allylcysteine levels	Inverse variance weighted	17	0.019351016	0.019585128	0.323129901	-0.019035835	0.057737867	1.019539461	0.981144203	1.059437246	56.9
LOAD	Campesterol levels	Inverse variance weighted	17	0.017886265	0.021893294	0.413942938	-0.025024591	0.060797122	1.018047183	0.975285928	1.062683297	56.9
LOAD	N2-acetyl,N6-methyllysine levels	Inverse variance weighted	17	0.023440536	0.023399538	0.316463342	-0.022422558	0.06930363	1.023717424	0.977826959	1.071761579	56.9
LOAD	Hexadecenedioate (C16:1-DC) levels	Inverse variance weighted	17	-0.013715957	0.021638545	0.526167715	-0.056127505	0.028695592	0.986377679	0.945418583	1.029111277	56.9
LOAD	Tetradecanedioate (C14-DC) levels	Inverse variance weighted	17	-0.004969947	0.025642654	0.846320262	-0.055229549	0.045289655	0.995042383	0.946267909	1.046330891	56.9
LOAD	Serine to threonine ratio	Inverse variance weighted	17	-0.020432965	0.020186178	0.311430221	-0.059997874	0.019131944	0.979774373	0.941766536	1.019316132	56.9
LOAD	Glycine to serine ratio	Inverse variance weighted	17	-0.01333951	0.020825972	0.521832746	-0.054158415	0.027479394	0.986749067	0.947282031	1.027860435	56.9
